# Preclinical Characterization of Antinociceptive Effect of Bergamot Essential Oil and of Its Fractions for Rational Translation in Complementary Therapy

**DOI:** 10.3390/pharmaceutics14020312

**Published:** 2022-01-28

**Authors:** Damiana Scuteri, Laura Rombolà, Michele Crudo, Chizuko Watanabe, Hirokazu Mizoguchi, Shinobu Sakurada, Kengo Hamamura, Tsukasa Sakurada, Paolo Tonin, Maria Tiziana Corasaniti, Giacinto Bagetta

**Affiliations:** 1 Pharmacotechnology Documentation and Transfer Unit, Preclinical and Translational Pharmacology, Department of Pharmacy, Health and Nutritional Sciences, University of Calabria, 87036 Rende, Italy; 2 Regional Center for Serious Brain Injuries, S. Anna Institute, 88900 Crotone, Italy; patonin18@gmail.com; 3 Preclinical and Translational Pharmacology, Department of Pharmacy, Health and Nutritional Sciences, University of Calabria, 87036 Rende, Italy; laura.rombola@unical.it (L.R.); michelecrudo@virgilio.it (M.C.); 4 Department of Physiology and Anatomy, Faculty of Pharmaceutical Sciences, Tohoku Medical and Pharmaceutical University, 4-4-1 Komatsushima, Aoba-ku, Sendai 981-8558, Japan; w-chizu@tohoku-mpu.ac.jp (C.W.); mizo@tohoku-mpu.ac.jp (H.M.); 5 First Department of Pharmacology Fukuoka, Daiichi College of Pharmaceutical Sciences, Fukuoka 815-8511, Japan; s-sakura@tohoku-mpu.ac.jp (S.S.); k-hamamura@daiichi-cps.ac.jp (K.H.); tsukasa@daiichi-cps.ac.jp (T.S.); 6 Department of Health Sciences, University “Magna Graecia” of Catanzaro, 88100 Catanzaro, Italy; mtcorasa@unicz.it

**Keywords:** bergamot essential oil, decolored fraction, deterpenated fraction, limonene, linalool, linalyl acetate, pain, formalin test, locomotor activity, inhalation route

## Abstract

Bergamot essential oil (BEO) is endowed with consistent and reproducible antinociceptive and anti-allodynic properties when administered via an inhalation route. However, the effects of its main constituents and of its decolored (DEC) and deterpenated (DET) fractions, which are enriched in limonene or in linalool and linalyl acetate, respectively, on spontaneous motor activity related to anxiety and on formalin-induced licking/biting biphasic behavior have never been investigated before. Therefore, the present research aims to characterize the role of BEO components on an experimental pain model that is relevant to clinical translation. Under our present experimental conditions, a paper filter disc soaked with different volumes of the phytocomplex and of its fractions that was applied at the edge of the observation chamber allowed the effects on the spontaneous motor activity and on the formalin-induced nocifensive response in ddY-strain mice to be studied. The present research demonstrated the effects of the DEC fraction of BEO on motor activity and on formalin-induced licking/biting behavior for the first time, proving that limonene is implicated in reduced motor activity and that it is important for the analgesic effect.

## 1. Introduction

The translational use of essential oils still requires preclinical validation in support of clinical efficacy and safety. However, this evidence has already been achieved for bergamot essential oil (BEO) [1]. BEO is produced from the cold pressing of the epicarp and the partly cold pressing of the mesocarp of fresh bergamot fruit (*Citrus bergamia* Risso et Poiteau), and it is composed of a volatile fraction (93–96%) that includes monoterpene and sesquiterpene hydrocarbons, of which one of the most important is limonene. Additionally, the volatile fraction includes oxygenated derivatives, such as linalool. Finally, there is a nonvolatile fraction (4–7%) that is made up of waxes, polymethoxylated flavones, coumarins, and psoralens such as bergapten (5-methoxypsoralen) and bergamottine (5-geranyloxypsoralen) [2,3]. BEO has shown strong preclinical evidence of analgesic activity both in nociceptive and neuropathic pain models. In particular, it is endowed with anti-nociceptive efficacy, as observed in the capsaicin test, and has been proven to involve the peripheral opioid receptors and to enhance the analgesia afforded by morphine [4]. This is fundamental because opioids are normally ineffective or poorly effective in managing neuropathic pain [5] as well as pain that is central in origin [6]. Moreover, it exerted an anti-allodyinic effect in a partial sciatic nerve ligation (PSNL) model involving the extracellular signal-regulated kinases (ERK) [7]. Incidentally, the anti-neuropathic pain effect that was observed in this model was also demonstrated for long-lasting continuous administration via an osmotic pump, mimicking in-clinic chronic pain treatment [8]. Among behavioral pain models, the formalin test is particularly worthy of note due to its biphasic nature, as it includes both a local nociceptive response and a secondary central sensitization that is peculiar of chronic pain [9] that is experienced and inappropriately treated in real-world settings [10,11,12]. The inhalation of BEO was proven to exert analgesia in the formalin test [13] as well as an anxiolytic effect through aromatherapy in clinic [14]. However, the effects of the whole phytocomplex and of its main pharmacologically active components, i.e., limonene, linalool and linalyl acetate, still need to be elucidated. To achieve this, the present research investigates how the inhalation of different volumes of the latter components of BEO and of its decolored (DEC) and deterpenated (DET) fractions, which are enriched in limonene or in linalool and linalyl acetate, respectively, affect spontaneous motor activity related to anxiety and formalin-induced licking/biting biphasic behavior. All the fractions tested were defurocoumarinized to avoid phototoxicity.

## 2. Materials and Methods

### 2.1. Animals

Male ddY mice (Japan SLC, Hamamatsu, Japan) that were 23–25 g in weight at the beginning of the experiments were used. Animals were housed individually with a 12 h light/dark cycle, room temperature 23 °C, and 50–60% relative humidity and were provided with food and water ad libitum. All of the experiments followed the Guidelines on the Ethical Standards for Investigation of Experimental Pain in Animals, and they are approved by the Committee of Animal Care and Use of Tohoku Medical and Pharmaceutical University (Project PADOEB, approved on 8 January 2014) in order to minimize animal suffering and to ensure the only the number of animals necessary to obtain reliable results were used. A total of *n* = 8 was used for each experiment according to the G*power sample size, which was determined based on previous literature [13].

### 2.2. Experimental Procedure

**Figure 1:**
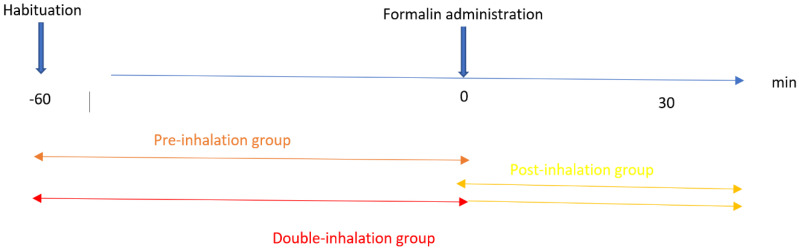
Protocol for bergamot essential oil (BEO) inhalation and that of its fractions with respect to the formalin test.

Mice were habituated in a transparent cage (22.0 cm × 15.0 cm × 12.5 cm), which was subsequently used as an observation chamber, and the cage was covered with another plexiglass cage to prevent the inhaled BEO from leaking. Accordingly, a dry (control) filter paper disc or one that had been soaked with different volumes of BEO or of its fractions was applied to the top of the cage for 5 min before the beginning of the observation. Three different inhalation times were investigated (pre-, post-, and double inhalation). The pre-inhalation consisted of pre-treatment with BEO or its fractions for 1 h during the habituation period, after which formalin was injected intraplantarly (i.pl.). In the case of post-inhalation, inhalation started immediately after the i.pl. injection of formalin and lasted for the whole duration of the formalin test. The overall motor and analgesic effects were assessed in the double inhalation group, in which the inhalation started as a pre-treatment for 1 h during the habituation period and continued immediately after formalin administration for the whole formalin test. The purpose of the double inhalation procedure was to assess the total effects of the pre- and post-inhalation treatments. Spontaneous motor activity was assessed by monitoring the periods of activity/inactivity in the animals for 60 min. The formalin test consisted of an i.pl. injection of 20 μL of formalin (2% in saline) through a microsyringe with a 26-gauge needle. The behavioral pain indicator was monitored by determining the licking/biting time using a handheld stopwatch at intervals of 5 min. The most important time points were the early phase, taking place 10 min (0–10 min) after formalin administration, and the late phase, which lasted for 20 min (10–30 min) following formalin administration. The first phase is a nociceptive phase, while the second involves central sensitization mechanisms, and between the first two phases, there is an interphase characterized by reduced licking/biting [15,16]. The experimental protocol is summarized in Figure 1.

### 2.3. Phytocomplexes and Components

BEO and its fractions were provided by “Capua Company1880 S.r.l.,” Campo Calabro, Reggio Calabria (Italy). Based on the chromatographic analysis reported in the certificate of analysis, the batch of BEO that was used was composed of D-limonene (39.60%), linalyl acetate (31.09%), linalool (9.55%), and bisabolene (0.45%), with a bergapten content of 2783,33 ppm and a bergamottin content of 26,931 ppm. The decolored fraction (DEC; batch 14/01670—103/2014) differs from the whole phytocomplex due to its higher levels of D-limonene (44.00%) and lower levels of linalyl acetate, linalool, and bisabolene (28.23%, 4.45%, and 0.31%, respectively). Compared to the BEO, the deterpernated fraction (DET; batch 14/01671—103/2014) is characterized by lower levels of D-limonene (7.58%) and higher levels of linalyl acetate (44.44%), linalool (39.83%), and bisabolene (0.61%). Limonene (CAS No. 5989-27-5) was bought from Sigma-Aldrich (Sigma–Aldrich Chemical Co., St. Louis, MO, USA), and linalyl acetate (CAS No. 115-95-7) and linalool (CAS No. 78-70-6) were provided by TCI (Tokyo Chemical Industry Co. Ltd., Tokyo, Japan). The phytocomplexes and their components were diluted in Jojoba oil (CAS No. 61789-91-1, Wako Pure Chemical Industries, Ltd., Osaka, Japan).

### 2.4. Reagents

Formalin (36% solution; Kanto Chemical, Tokyo, Japan) was diluted in saline solution to obtain a final concentration of 2% immediately before use.

### 2.5. Statistical Analysis

The results are presented as mean ± S.E.M. of the minutes of locomotor activity and of the seconds of licking/biting and were assessed for differences by One- or Two-way ANOVA followed by Bonferroni’s test. Values of *p* < 0.05 are considered statistically significant.

## 3. Results

### 3.1. Effects on Spontaneous Motor Activity

**Figure 2:**
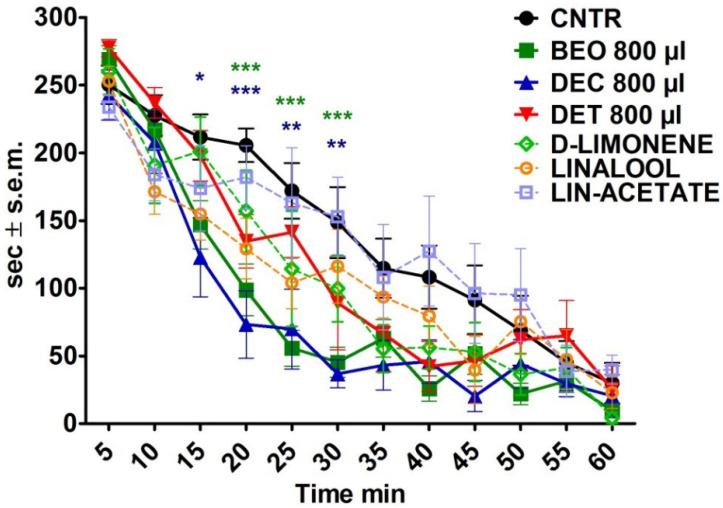
Time course of the effects of the bergamot essential oil (BEO), of the decolored (DEC) and deterpenated (DET) fractions, and of D-limonene, linalool, and linalyl acetate on locomotor activity in seconds (sec) at 5 min intervals. Two-way ANOVA followed by Bonferroni test; *n* = 8, * *p* < 0.05, ** *p* < 0.01, *** *p* < 0.001.

The exposure of the mice to BEO and to its DEC fraction produced a significant reduction in the locomotor activity with a progressive increase in the inactivity time, which occurred in a volume-dependent manner. A significant reduction in locomotor activity was already observable15 min after treatment with 800 µL of the DEC fraction (*p* < 0.05). Moreover, after 20 min of treatment, there was a marked reduction in motor activity that was induced by the inhalation of 800 µL of both BEO and the DEC fraction (*p* < 0.001). A significant reduction in the overall activity compared to the control was also observed after 25 and 30 min of exposure to BEO (*p* < 0.001) and its DEC fraction (*p* < 0.01). The inhalation of the DET fraction as well as the inhalation of D-limonene, linalool, and linalyl acetate when administered individually, only caused a slight reduction in the overall activity of the animals and was not significant at the considered time intervals. The results are presented in Figure 2.

**Figure 3:**
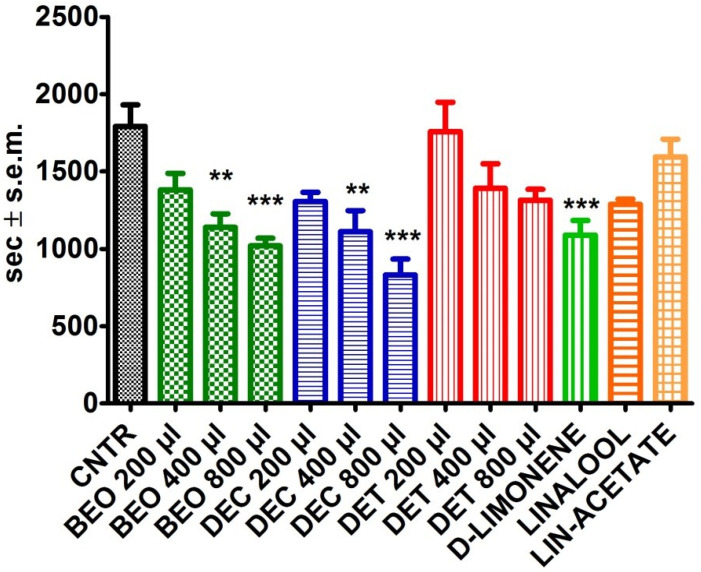
Effects of bergamot essential oil (BEO), of the decolored (DEC) and deterpenated (DET) fractions, and of D-limonene, linalool, and linalyl acetate (expressed in seconds (sec)) on locomotor activity after one hour of observation. One-way ANOVA followed by Bonferroni test; *n* = 8, * *p* < 0.05, ** *p* < 0.01, *** *p* < 0.001.

Evaluating the overall motor activity of the animals throughout the observation period, a significant reduction in activity time following the inhalation of 400 µL (*p* < 0.01) and 800 µL (*p* < 0.001) of BEO and DEC can be observed (Figure 3). No significant effects were observed for D-limonene. However, the amount of D-limonene (352 µL) present in 800 µL of the DEC fraction produced a significant reduction in the overall activity (*p* < 0.001) (Figure 3). Volumes of 320 µL of linalool and 356 µL of linalyl acetate, which correspond to the levels present in 800 µL of the DET fraction, do not produce significant changes in the overall activity time of the animals (Figure 3).

### 3.2. Analgesic Effects of BEO Enriched Fractions on Formalin Test

**Figure 4:**
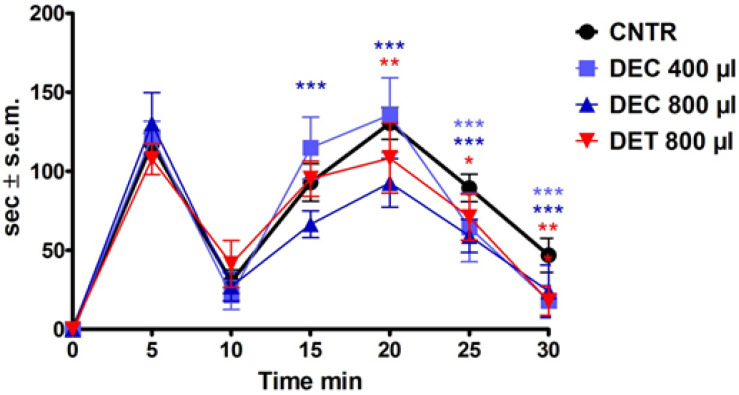
Effects of bergamot essential oil (BEO) and of its decolored (DEC) and deterpenated (DET) fractions on 2% formalin-induced licking/biting behavior expressed in seconds (sec). Two-way ANOVA followed by Bonferroni test; *n* = 8, * *p* < 0.05, ** *p* < 0.01, *** *p* < 0.001.

The inhalation of BEO, which was administered immediately after the i.pl. injection of formalin, significantly reduced the licking/biting time in a volume-dependent manner [13]. Similarly, 800 µL of the DEC fraction induced a significant reduction in the licking/biting behavior during the second phase of the nociceptive response induced by formalin (*p* < 0.001); in particular, this reduction was recorded during the time interval between 15 and 30 min following the injection (Figure 4).

**Figure 5:**
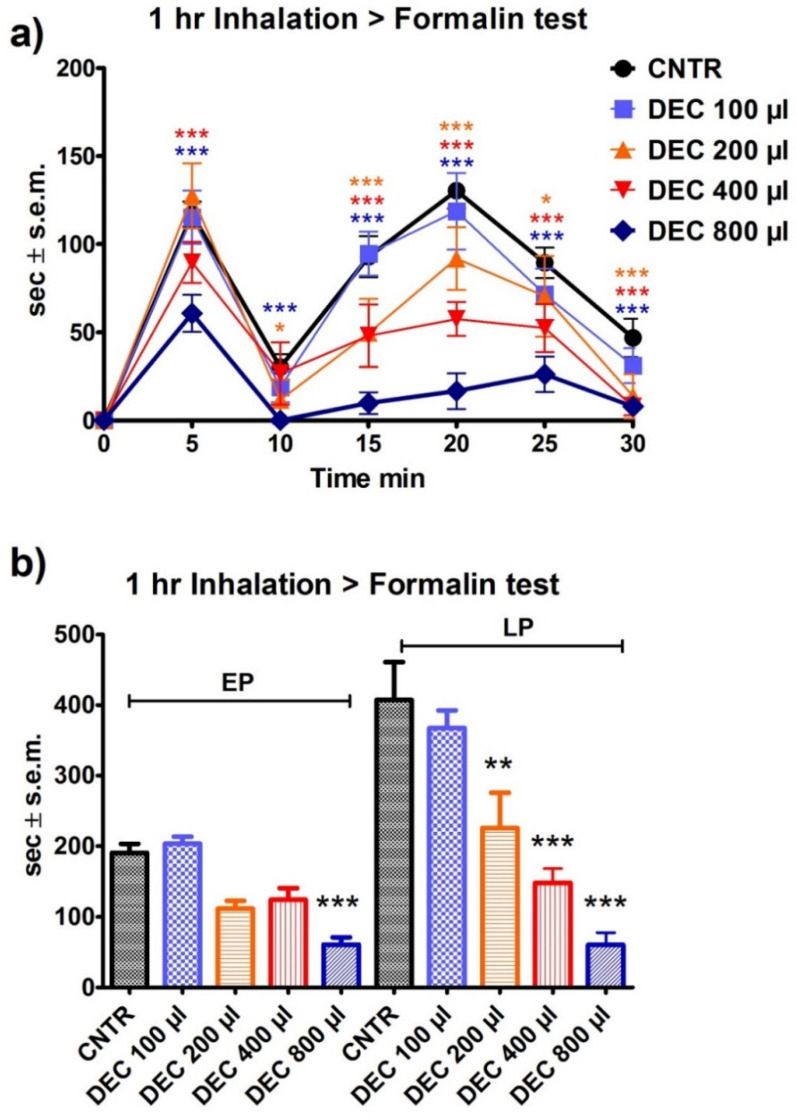
Effect of the one-hour pre-treatment with the decolored fraction (DEC) of bergamot essential oil (BEO) on 2% formalin-induced licking/biting behavior. (**a**) Time course based on seconds (sec) of licking/biting recorded at intervals of 5 min and expressed as time in sec ± S.E.M. (**b**) Overall licking/biting time expressed as time in sec ± S.E.M. during the first phase (EP, 0–10 min) and the second phase (LP, 10–30 min). Two-way ANOVA followed by Bonferroni test; *n* = 8, * *p* < 0.05, ** *p* < 0.01, *** *p* < 0.001.

The pre-treatment of the inhalation of 200, 400, and 800 µL of DEC was shown to induce a significant reduction in the overall licking/biting behavior in a volume-dependent manner, both during the first as well as during the second phase of the nociceptive response induced by the i.pl. injection of formalin (200 µL: *p* < 0.01; 400 and 800 µL: *p* < 0.001) (Figure 5a,b).

**Figure 6:**
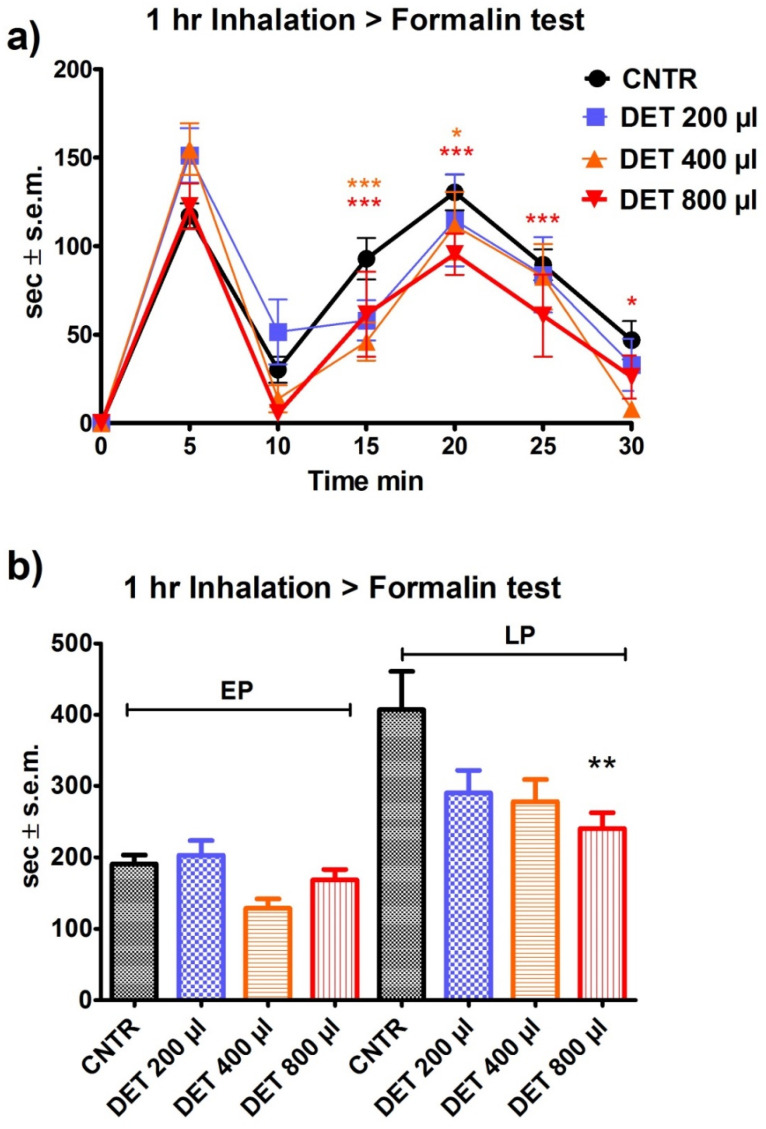
Effect of the one-hour pre-treatment with the deterpenated fraction (DET) of bergamot essential oil (BEO) on 2% formalin-induced licking/biting behavior. (**a**) Time course based on seconds (sec) of licking/biting recorded at intervals of 5 min and expressed as time in sec ± S.E.M. (**b**) Overall licking/biting time expressed as time in sec ± S.E.M. during the first phase (EP, 0–10 min) and the second phase (LP, 10–30 min). Two-way ANOVA followed by Bonferroni test; *n* = 8, * *p* < 0.05, ** *p* < 0.01, *** *p* < 0.001.

The 1 h pre-treatment with DET proved to be less effective in reducing the nociceptive response induced by formalin. In fact, a significant reduction (*p* < 0.01) in the licking/biting behavior was observed during the second phase of the nociceptive response to formalin following exposure to 800 µL of DET and not at lower volumes (Figure 6a,b).

## 4. Discussion

The present research demonstrates the analgesic efficacy of the DEC and DET fractions of BEO administered via an inhalation route in 2% formalin-induced biphasic licking/biting behavior for the first time. The analgesic efficacy of BEO inhalation is higher than that observed for its fractions, as previously demonstrated [13], but at the highest volume (800 µL), the DEC fraction is as effective as comparable volumes of the whole phytocomplex. This does not occur for the DET fraction, which is less effective, even at the highest volume. Moreover, BEO and the DEC fraction administered via an inhalation route in the volumes of 400 and 800 µL provide a comparable statistically significant spontaneous motor activity decrease effect. The latter reduction exerted by 400 µL of BEO and the DET fraction is paralleled by pure limonene. Therefore, due to the effect of the whole phytocomplex and of the DEC fraction on motor activity and on the formalin-induced nociceptive response, limonene is important for the analgesic effect and is implicated in reduced motor activity. The latter is related to an anxiolytic-like effect. In particular, the effect of BEO in widely used behavioral tasks for the assessment of anxiolytic and antidepressive properties of compounds, i.e., the open field, the elevated plus-maze and the forced swimming (FST) tasks, proves increase in the immobility time in open field and in the forced swimming tests [17]. The latter observation supports evidence of having BEO anxiolytic-like effects, and that limonene plays an important role since high locomotory activity is associated with attempts to escape, resulting in being a remarkable behavioral indicator of anxiety [18]. Incidentally, these effects have been demonstrated to be devoid of the sedation that is typical of benzodiazepines [19] and to involve serotonergic neurotransmission [20] that is separate from the possible role of the glutamatergic and endocannabinoid systems [21,22,23]. This is fundamental since the glutamatergic [24,25] and serotonergic [26] mechanisms are significantly involved in aging and neurodegeneration and, thus, they are also involved in the mechanism of action of rapid acting antidepressants [27]. This finding could break new ground for the treatment of neuropsychiatric symptoms [28]. This deepening of the characterization of the neuropharmacological profile of BEO is fundamental to elucidate all of its properties since BEO may provide an effective option for the management of agitation that is closely connected to under-treated pain [29] and, unfortunately, that is managed with antipsychotics, doubling the risk of death caused by cardiocerebrovascular accidents in the elderly [30], something that is even more relevant during the pandemic [31]. In fact, BEO has been engineered in the pharmaceutical form of a nanotechnological cream for its delivery [32], and the clinical trial for proof of concept in severe dementia (NCT04321889) [33] is ongoing. Future studies need to clarify the role of the single components since the whole phytocomplex is endowed with the highest amount of pharmacological activity.

## Data Availability

Data are contained within the article.
